# Design Ideas for Inpatient Stroke Rehabilitation Facilities: Living Lab Findings

**DOI:** 10.1177/19375867251343910

**Published:** 2025-07-22

**Authors:** Ruby Lipson-Smith, Aaron Davis, Marcus White, Luis Pflaumer, Julie Davey, Leonid Churilov, Anna Fox, Natalie Pitt, Ciara Shiggins, Juan Pablo Saa, Mark Lam, Julie Bernhardt, on behalf of the NOVELL Collaboration

**Affiliations:** 1 56369 The Florey Institute of Neuroscience and Mental Health, Heidelberg, Australia; 2 The MARCS Institute for Brain, Behaviour and Development, 6489 Western Sydney University, Westmead, Australia; 3 UniSA Creative, 1067 University of South Australia, Adelaide, Australia; 4 Centre for Design Innovation, 3783 Swinburne University of Technology, Hawthorn, Australia; 5 Stroke survivor co-researcher, Melbourne, Australia; 6 Department of Medicine, University of Melbourne, Melbourne, Australia; 7 STH Health Architecture, Australia; 8 Centre of Research Excellence in Aphasia Recovery and Rehabilitation, Melbourne, Australia; 9 School of Health Sciences, The University of East Anglia, Norwich, UK; 10 Melbourne School of Design, 2281 The University of Melbourne, Parkville, Australia; 11 School of Design and Architecture, 3783 Swinburne University of Technology, Hawthorn, Australia

**Keywords:** living lab, co-design, co-creation, evidence based design, stroke, rehabilitation, hospital design, built environments

## Abstract

**Objectives:**

To provide actionable, co-designed ideas for how to optimize the built environment and service of inpatient stroke rehabilitation facilities.

**Background:**

Input from diverse stakeholders is needed to ensure that stroke rehabilitation spaces address the unique learning and practice needs of users. In this paper, we report the first phase of the Neuroscience Optimized Virtual Environments Living Lab (NOVELL) Redesign project.

**Method:**

We engaged with key stakeholders across: (1) Four co-design workshops (*n* ranged between 23 and 31 people per workshop including stroke survivors, clinicians, and designers) to generate ideas for design innovation; (2) a workshop with a healthcare architecture firm responding to these ideas; and (3) an online prioritization task to rank outcomes from previous workshops.

**Results:**

Outputs included: (1) A framework of objectives describing what is important in stroke rehabilitation environments and services; (2) 28 actionable design ideas for achieving these objectives; (3) 10 scenarios that integrate these design ideas and objectives to describe a speculative, visionary stroke rehabilitation facility; and (4) prioritization of these scenarios. Key scenarios included: Bedrooms that achieve the benefits of both a single and shared room; environments/services that allow stroke survivors access to appropriate levels of risk; and therapy spaces that provide supported challenge and real-world practice.

**Conclusions:**

We identified opportunities for innovation that bring service design and architectural design together symbiotically. The interdisciplinary methods—combining co-design, Design Thinking, Speculative Futures, and Multi-Attribute Evaluation within a Living Lab framework—were successful in generating collaborative, actionable, and visionary design ideas.

The incidence of stroke is increasing worldwide (Thayabaranathan et al., 2022). Many stroke survivors require a period of inpatient rehabilitation to re-learn the skills and abilities impacted by their stroke before being discharged to the community (Stroke Foundation, 2023). Inpatient stroke rehabilitation facilities are essential, but under-researched healthcare environments and are rarely purpose-built for rehabilitation (Lipson-Smith et al., 2021). The unique needs of stroke survivors make these environments an important target for innovation. Rehabilitation stays are significantly longer than on acute wards, extending to weeks or months (Australisian Rehabilitation Outcomes Centre [AROC], 2020). Stroke can impact many aspects of physical and cognitive functioning—including walking and arm use, and speech and communication—and can cause extreme fatigue and changes to mood and motivation. Guidelines recommend therapy schedules and daily routines that provide sufficient rest time while keeping survivors out of their beds as much as possible in order to promote the activity and practice that facilitate neuroplastic repair and help people to re-learn the skills and abilities impacted by stroke (Stroke Foundation, 2024), but, despite this, many survivors are inactive and in their bedrooms most of the day (Sjöholm et al., 2014; West & Bernhardt, 2012). Innovation of stroke rehabilitation environments requires a fundamental rethink of the relationship between service models and architectural spaces (Saa et al., 2023).

Healthcare facility design is complex. Stakeholders include healthcare users, healthcare workers, architects and designers, policy makers, building code authorities, and healthcare planners, among others. Innovation in healthcare facility design is often hindered by insufficient collaboration and communication between stakeholders and a gap between design research and practice (Halawa et al., 2020; Hall et al., 2017; Hamilton, 2015; Sal Moslehian et al., 2020, 2023). Collaboration efforts and attempts to bridge the research-practice gap are frustrated by the challenge of translating traditional research findings into tangible, actionable outcomes that can be applied by designers (Carthey, 2020). There is a need for new models of collaborative, visionary design that focus on actionable design solutions.

In 2019, we established a Living Lab to address the challenge of developing innovations for sub-acute inpatient stroke rehabilitation built environments and services, including the building, interiors, and the model of care. This project works with the assumption that, as in all complex healthcare contexts, the service and built environment of inpatient stroke rehabilitation are inextricably linked, hence making the dual focus an essential part of this research (Bernhardt et al., 2022). In brief, the project combines processes from co-design, design science, and neuroscience and has brought together stroke survivors, clinicians, academics, architects and designers, health care planners, and policy makers as “co-researchers” to develop a new, innovative best-practice standard for stroke rehabilitation environments.

*In 2019, we established a Living Lab to address the challenge of developing innovations for sub-acute inpatient stroke rehabilitation built environments and services, including the building, interiors, and the model of care.*

In previous work, Value-Focused Thinking (Keeney, 1992) was used to create a framework of what is important in the design of stroke rehabilitation facilities (Lipson-Smith et al., 2019). In this framework, the most important criteria—described in the Value-Focused Thinking approach as “fundamental objectives” (Keeney, 1992)—were to provide a safe, efficient environment that maximizes opportunities for stroke survivor activity and practice (physical, cognitive, and social), sleep and rest, and fosters emotional well-being (see Figure 1). Fourteen additional criteria, or “means objectives,” were defined as being instrumentally important to achieving these goals (Lipson-Smith et al., 2019). In this present manuscript, we describe the first phase of our project, the “concept design” phase, in which we aimed to build on these objectives and generate specific, actionable, and solution-focused ideas for how to optimize the built environment and the service for inpatient stroke rehabilitation. These ideas were then organized and prioritized in collaboration with stakeholders, providing the basis of an evidence-based “design brief” and framework for prototyping and testing in subsequent phases of the Living Lab project.

**Figure 1.**
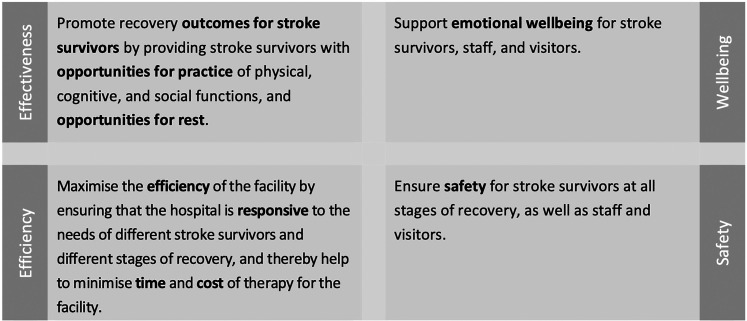
The four fundamental objectives for inpatient stroke rehabilitation environments: Effectiveness, efficiency, wellbeing, and safety. Note: These fundamental objectives were first defined in a previously published framework of what is important in inpatient stroke rehabilitation environments (Lipson-Smith et al., 2019).

## Methods

**Figure 2.**
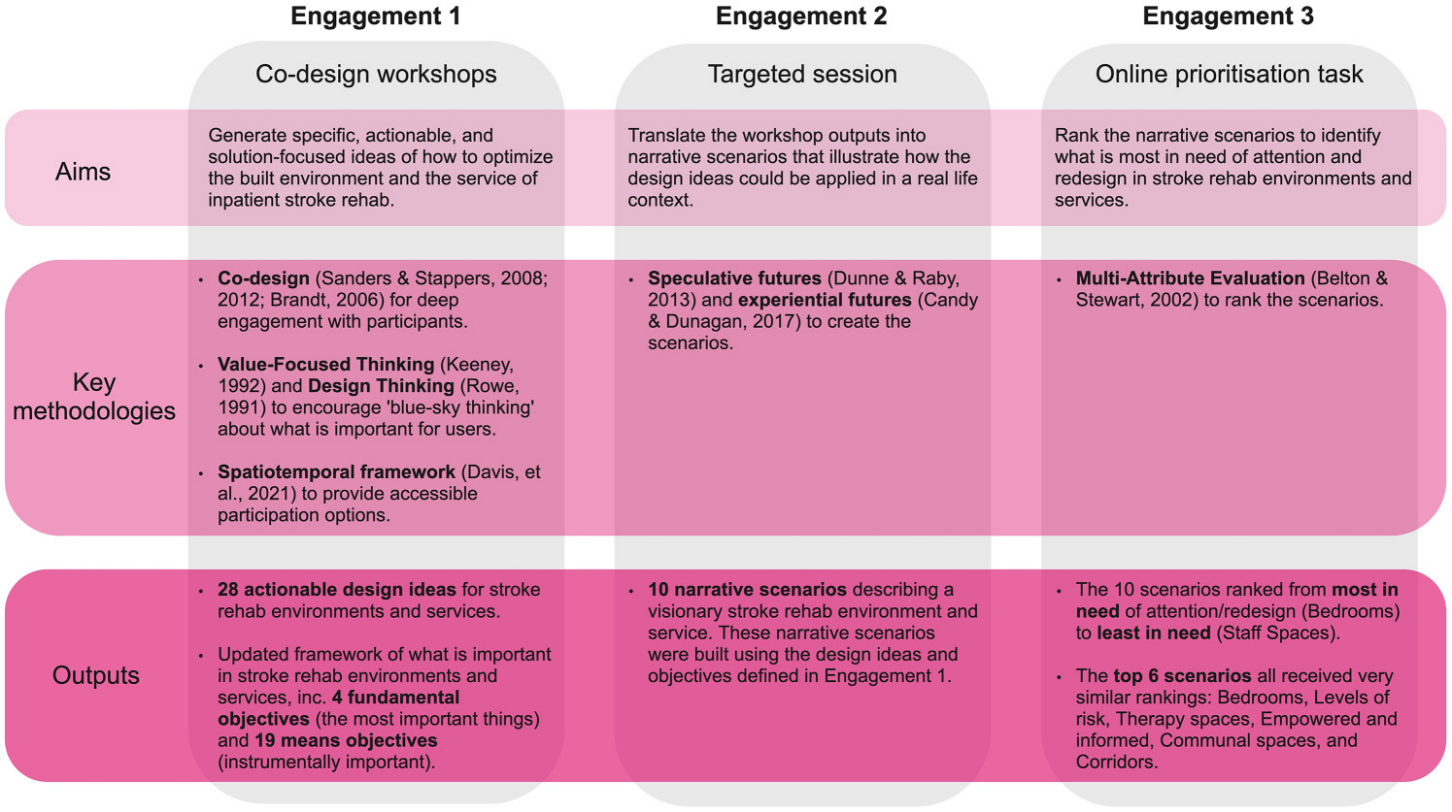
Summary of the aims, methods, and outputs of the three engagements in this study.

This first phase of the project comprised three distinct engagements with participants (see Figure 2). (1) Four half-day co-design workshops in 2020 with a highly diverse group of stakeholders, (2) a target engagement with an experienced healthcare architecture firm, and (3) an online prioritization task. Ethics approval was received from the Ethics Committee of one of the project partners.

### Co-Researchers

In line with the principles of a Living Lab, where public-private-people partnerships come together to create, prototype, validate and test ideas in real-life settings (Leminen et al., 2012), workshop attendees were engaged as “co-researchers” not “participants.” This confirmed the partnership-nature of their engagement in the project, and ensured co-researchers saw themselves as actively contributing to solutions rather than as a passive source of information (Malterud & Elvbakken, 2020). All the co-researchers engaged throughout the project (*n* = 131) are acknowledged in the authorship of this paper (see Supplemental Material 1). For this first phase, focused on creating and prototyping, potential attendees were identified through existing networks. Target groups included stroke survivors, rehabilitation clinicians and healthcare professionals, healthcare architects and other designers such as wayfinding and accessibility consultants, and academics. Attendees were selected based on their field of work, familiarity with healthcare environments and stroke rehabilitation, and availability.

### Engagement 1: Co-Design Workshops

Co-design focuses on deep engagement with participants by reducing power imbalances, and valuing pluralistic perspectives, researching “with” rather than “on,” “about,” or “for” participants (Sanders & Stappers, 2008). For each workshop, a series of custom-made tasks were developed based on the principles of co-design outlined by Sanders and Stappers (2012) and Brandt (2006). Activities were designed to specifically help participants shift from sharing explicit knowledge to expressing their tacit and latent knowledge through structured creative-practice activities (Sanders & Stappers, 2012). In this first stage of the project, workshop facilitators encouraged attendees to take a “blue sky thinking” approach, and not be constrained by current norms in stroke rehabilitation, or by perceived financial costs as these would be considered in subsequent analytically focused stages of the living lab.

The co-design workshops were initially planned as four half-day face-to-face gatherings, but due to public activity restrictions associated with coronavirus disease 2019, these were redesigned so that they could be held online using video conferencing (Zoom) and a shared online workspace (Miro). Facilitators were experienced in co-design methods and attended a training session prior to each workshop in which they worked through each of the workshop activities.

Workshop materials and activities were prepared in consultation with a speech pathologist with expertise in the communication and cognitive challenges associated with stroke. Importantly, the workshops were not simply translated into an “online version of a face-to-face workshop,” but instead, redesigned to respond to the various technologies and approaches available. As part of this redesign, a spatiotemporal framework for co-design (Davis et al., 2021), was used to structure opportunities for co-researchers to contribute in different ways, including through live online engagements, asynchronous preparation and follow up activities, and hybrid digital/physical tasks. The speech pathologist worked with stroke survivors with particular communication needs (e.g., aphasia) to support them to be included in, and contribute to, the workshop sessions. In some instances, this included undertaking activities with stroke survivors in advance of the workshop sessions, holding asynchronous one-to-one activity sessions, or acting as a supportive communication partner.

Before each workshop, attendees were provided with a briefing pack containing background information on the project, links to video-based information about stroke and current stroke rehabilitation processes, instructions on how to prepare for the workshop, a guide for Zoom and Miro, the workshop agenda, and a glossary of key terms. The briefing pack also provided a link to a series of training videos and practice activities for attendees to familiarize themselves with the technologies being used in the workshop sessions. In addition, participants were offered the option of one-on-one online briefing with a member of the research team prior to each workshop.

#### Workshop Activities

Each of the four workshops in this initial engagement addressed a different series of interrelated topics (see Table 1). These topics had been identified as opportunities for innovation in prior research (Lipson-Smith et al., 2019). We used Value-Focused Thinking and Design Thinking approaches and generative research activities to help attendees co-create both speculative and pragmatic ideas (Keeney, 1992; Rowe, 1991). Value-Focused Thinking encourages a focus on what is important, or of value, before thinking about how these important things could be achieved (Keeney, 1992). Throughout the workshop activities, attendees were supported to generate highly speculative ideas, and to then translate these into specific, actionable, solution-focused outputs that could be operationalized into tangible or experiential designs for inpatient stroke rehabilitation. For example, “access to practice outside of formal sessions*”* or “socialisation should be a choice–some want less isolation, some don’t*”*, were actionable outputs generated in the workshops that had potential design, and/or service-based solutions specific to stroke rehabilitation. Stand-alone statements that did not imply a solution, such as “I don’t want to feel bored”, or statements of generic architectural principles, such as “I want good lighting”, were followed-up by further prompts from facilitators to explore how these thoughts might be actioned in a stroke-specific context through the explication of first-person scenarios.

*Attendees were supported to generate highly speculative ideas, and to then translate these into specific, actionable, solution-focused outputs that could be operationalized into tangible or experiential designs for inpatient stroke rehabilitation.*

**Table 1.** The Topics of the Four Online Workshops.

	Workshop topic	Areas explored^a^
Workshop 1	Responding to human needs: Designing for rehabilitation now and into the future	Adaptability Versatility Multipurpose circulation spaces
Workshop 2	Blurring boundaries: Establishing connections between rehabilitation spaces and their social and environmental context	Community integration Aesthetics Outdoor & green space Indoor environmental quality
Workshop 3	Navigation: Finding our way in complex environments	Personal control over the space Legibility Accessibility Sightlines
Workshop 4	Safety: Designing safe spaces that promote rehabilitation	Technology Safety guidelines (inc. behavioral safety and risk management) Manual handling

^a^ These 14 criteria were described in Lipson-Smith et al. (2019) as instrumentally important “means objectives” for optimal inpatient stroke rehabilitation environments.

To create a collaborative environment online and to maximize opportunities for each participant to make contributions, the workshops were structured with minimal “whole group” time. The introduction to the workshop activities, and context setting was conducted asynchronously via the briefing pack process. This meant that upon joining each workshop, attendees were welcomed and given a brief summary of the session before being moved into pre-determined interdisciplinary working groups of four to five individuals, accompanied by a facilitator. Each group was allocated to a “breakout room” on Zoom, enabling conversations to take place at a small group scale. At key points during the workshops, all attendees re-joined the larger group to share a summary of their work, but the majority of time was spent working in small groups to increase each individual's opportunity for contributing. This approach was further enhanced by parallel working processes, where the game-based activities on the Miro board allowed attendees to work on the activities concurrently throughout the session rather than passively waiting to speak in a “turn-taking” environment (Brandt, 2006). All attendees were encouraged to continue adding to the Miro board after the conclusion of the workshop, allowing time for reflection and individual input not influenced by the group.

#### Co-Analysis

The principles of co-design were also incorporated in the analysis of the data. The task-based facilitation largely replaced the need for a traditional “scribe” and gave co-researchers the opportunity to ensure their thoughts and contributions were captured how they wished them to be. Workshop attendees were asked to undertake an initial co-analysis of the data that were being generated. In some instances, attendees were asked to individually identify what they saw as the most important or innovative outputs (the “highlights”) generated during each workshop. In other instances, power hierarchies were managed by asking attendees to collectively prioritise outputs using an eight-point agreement continuum, ranging from “veto, I will block this proposal” to “whole-hearted endorsement” (Brause, 2016, p. 90). All attendees were given four weeks access to the Miro boards after each workshop to continue adding any additional content, before the Miro boards were exported as PDFs and uploaded to NVivo 12 for data management and continued analysis (Lumivero, 2017).

An inductive thematic analysis was then undertaken using generic qualitative inquiry (Patton, 2015), whereby we identified recurring concepts—specifically, actionable outputs (design or service recommendations)—from the workshop outputs by allowing the data to speak for itself without any preconceived hypotheses. A member of the research team used open, or emergent, coding to group the actionable outputs (text and images) where they expressed similar information (Glaser & Strauss, 2017). This process was undertaken first with outputs that had been identified as “highlights” by co-researchers (see Supplemental Material 2), before integrating the remaining data. Codes were then reviewed by other members of the research team and similar codes were combined using axial coding (Corbin & Strauss, 2014) to describe a final set of clear, solution-focused, actionable outputs. These outputs were labeled according to whether they related to the built environment, technology, and/or the service.

#### Updating the Framework

The actionable outputs from the workshops were then mapped against the framework of fundamental and means objectives from previous work (Lipson-Smith et al., 2019) and reviewed by the team. Through this deductive process, the workshop outputs were used to confirm, augment, and expand the existing framework.

### Engagement 2: Creating Speculative Scenarios from the Workshop Findings

Following the workshops in Engagement 1, we worked with an experienced healthcare architecture firm to identify examples from past projects where the ideas generated in Engagement 1 might already have been demonstrated, and to explore how these could translate into future healthcare facilities. Outputs from the Engagement 1 workshops were grouped to describe different “scenarios” as part of a speculative futures process (Dunne & Raby, 2013), with each scenario illustrating how the workshop outputs could be translated into real changes and innovations in the built environment, technology, and service. The architects were then guided through an experiential futures activity to review the scenarios, construct new scenarios, and collaboratively explore and construct a series of possible futures including elements of “setting,” “scenario,” “story” and “stuff” (Candy & Dunagan, 2017). For each scenario, the attendees collected and contributed images and examples from practice where similar or analogous ideas had been explored, capturing how the concepts might have been reflected in architectural practice within and beyond healthcare projects.

### Engagement 3: Prioritizing the Scenarios via a Multi-Attribute Evaluation Panel

A panel of a purposive sample of attendees from the Engagement 1 workshops was formed. Specific people were invited to ensure equal representation from the different stakeholder groups. The Multi-Attribute Evaluation was completed online to review and prioritize the narrative scenarios (see Supplementary Material 3). Respondents could choose to complete the online task in their own time or with assistance via videoconference with a research team member.

The online task was designed and analysed following a Multi-Attribute Evaluation approach, whereby the scenarios were ranked by individual respondents according to predefined criteria (Belton & Stewart, 2002). Specifically, respondents were asked to rank the scenarios according to how in need they were of innovation, attention, and redesign, and to inform their ranking, they were asked to consider (1) the extent to which each scenario relates to, or has the potential to address, the things that are fundamentally important in stroke rehabilitation—efficiency, activity and rest, wellbeing, and safety (Lipson-Smith et al., 2019) and (2) the extent to which, in their personal experience of stroke rehabilitation environments, each of the scenarios were already well-realized in rehabilitation design. Individual rankings were combined using a graph-theory-based algorithm to produce an aggregated ranking of all scenarios (Utley et al., 2007). The group ranking algorithm arranges the scenarios in decreasing order of preference, based on three independent indices where the second and third indices are used to break the ties between different scenarios of the first index, should ties occur (Utley et al., 2007). Respondents were also given the opportunity to explain their rankings in short answer format.

## Results

The results of the workshops are reported in three parts: (1) Outputs from Engagement 1, including the actionable design ideas, and the updated framework of what is important in stroke rehabilitation environments and services; (2) a description of the scenarios developed following Engagement 2 that describe a speculative, visionary stroke rehabilitation environment and service; and (3) the prioritization of these scenarios (Engagement 3).

### Engagement 1: Outputs from the Co-Design Workshops

A total of 43 co-researchers were involved in the Engagement 1 workshops (Table 2 shows their primary expertise); 18 of whom had dual or previous expertise in two or more of the target disciplines (Table 3). Involvement across the series of workshops depended on each person's preferences and expertise, so some co-researchers attended every workshop while others only attended workshops that aligned with their interests. There were 26 attendees at workshop 1, 31 at workshop 2, 30 at workshop 3, and 23 at workshop 4.

**Table 2.** The Number of Co-researchers at the Engagement 1 Workshops.

Discipline	Healthcare environments	Stroke rehabilitation	Total
Academic	5	2	7
Architect/designer	15	N/A	15
Clinician	N/A	13	13
Stroke survivor	N/A	8	8
Total	20	23	43

*Note*: Co-researchers are listed by their primary expertise only

**Table 3.** The Engagement 1 Workshop co-Researchers Dual or Previous Expertise.

Type of dual expertise	Number of co-researchers
Academic + Architect/Designer	6
Clinician + Academic	9
Clinician + Architect/Design	1
Stroke Survivor + Healthcare	2
Total	18

#### Actionable Ideas for Optimizing Stroke Rehabilitation Environments and Services

**Figure 3.**
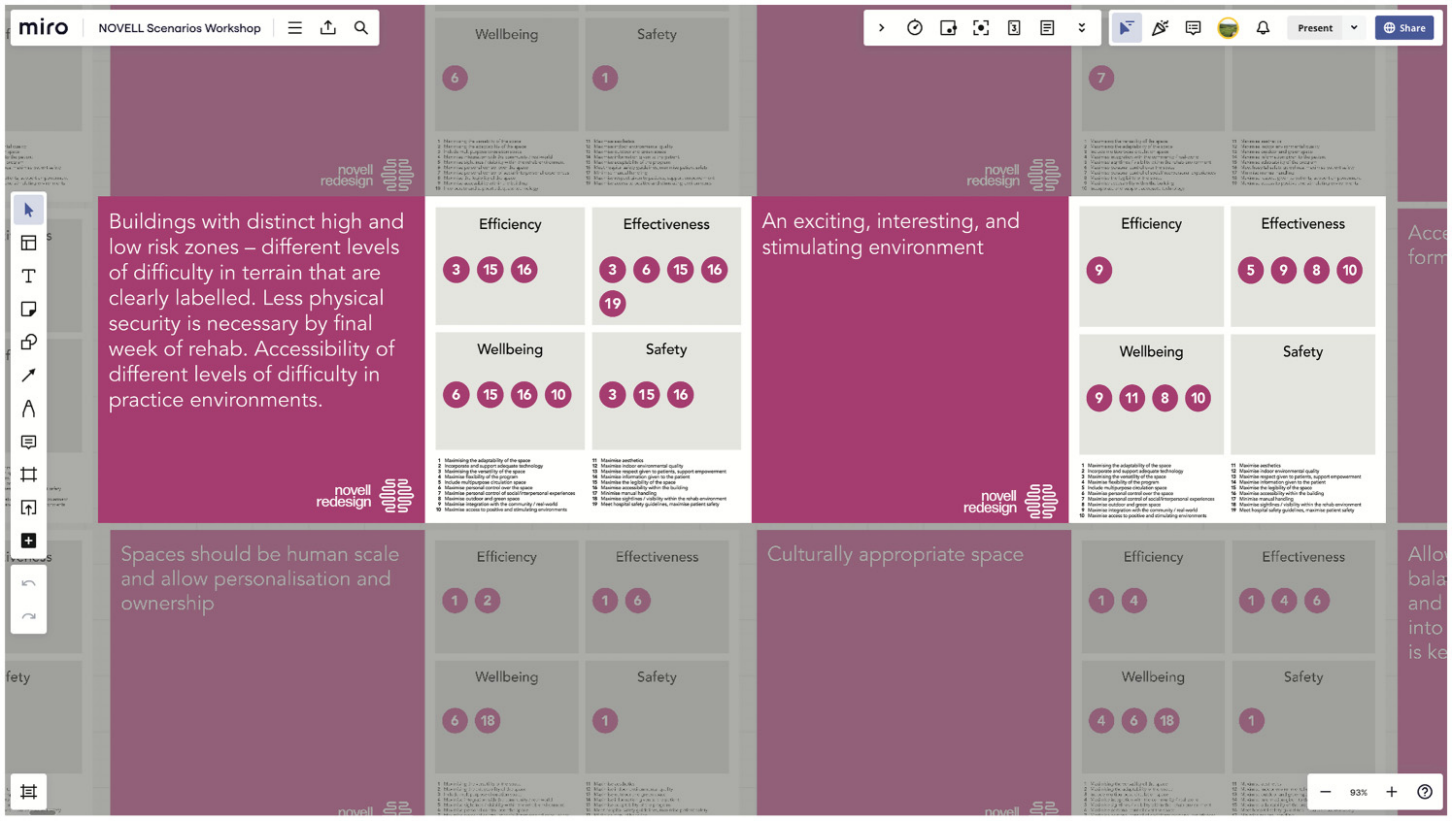
A screenshot of the shared online workspace (Miro board), zoomed in to show the virtual cards for two of the actionable design suggestions that were identified in the workshops. These cards, which were shown to attendees, included a description of the suggestion, mapped against the fundamental and means objectives for stroke rehabilitation environments and services that it would help to achieve. The full set of 28 virtual cards are provided in Supplementary Material 3, where they can be viewed in detail.

Twenty-eight distinct, actionable suggestions were identified through co-analysis of the workshop content (see Table 4). Some of the suggestions were relevant mainly to the physical environment (e.g., “regular rest areas along journeys”), but most had implications for both the environment and the service (e.g., “stroke survivor allowed to choose therapy location,” “access to practice outside of formal therapy sessions,” and “socialisation as a choice–some want less isolation, some do not,” to name a few). Virtual cards were created for each of these 28 suggestions, including a description of the suggestion alongside the fundamental and means objective/s that it was proposed to help to achieve, thereby drawing an explicit link between the suggestion and the objectives (two of these cards are shown in Figure 3, see Supplemental Material 4 for the full set of 28 cards).

**Table 4.** The 28 Actionable Design Suggestions for Inpatient Stroke Rehabilitation Environments and Services Identified in the Engagement 1 Co-design Workshops, Listed in no Particular Order.

	Actionable design suggestion
1	An exciting, interesting, and stimulating environment.
2	Control over sensory intensity—too much going on is confusing and overwhelming for some.
3	Facilitate socialization—different levels of socialization at different stages of the rehab journey. Includes engaging with other patients and staff, as well as family and friends while in rehab. Learning about and sharing in others’ successes. Sharing a meal with family.
4	Acoustic control and privacy through active or passive systems.
5	Get sense of purpose by doing real things—Being or seeing home prompts thinking about independence. Integrate with real-world in final week. Real-world furniture. Hospital as real-world playground. But remember, real world may not have the equipment or support needed.
6	Access to practice outside of formal sessions.
7	Opportunity for individual creative expression.
8	Socialization as a choice—some want less isolation, some don’t.
9	Rehab should be warm, comfortable and inviting not cold, dark or clinical.
10	Practice Activities of Daily Living (ADLs) such as showering and dressing in real bedroom or bathroom—this is easiest in single bed rooms.
11	Multiple levels of orientation and navigation.
12	Sensory connection with nature (sun, air, etc.).
13	Hospital boundaries that don’t disrupt social connection.
14	Spaces should be human scale and allow personalization and ownership.
15	Enabling rest especially in bedrooms—space to retreat.
16	Support a range of experiences.
17	Choice within and between spaces. Boundaries between private and communal activities.
18	An adaptive and flexible space can still be nurturing.
19	Bedroom tailored to person—Giving authority to change, rearrange and take ownership of bedroom and communal spaces. Spaces for me, my space, ownership.
20	Peaceful and or spiritual spaces—space to relax.
21	Environment that can change across a person's stay—support different models of care at different stages.
22	Need for intimacy—Privacy in activities to maintain dignity.
23	Buildings with distinct high and low risk zones—different levels of difficulty in terrain that are clearly labeled. Less physical security is necessary by final week of rehab. Accessibility of different levels of difficulty in practice environments.
24	Culturally appropriate space.
25	No “out of bounds” areas—if patients can access it, they can be there.
26	Patient allowed to choose therapy location.
27	Regular rest areas along journeys.
28	Allow people to fail safely—balanced risk leads to learning and recovery. Incorporate risk into the hospital, information is key. The right to fail.

#### The Updated Framework of What is Important in Stroke Rehabilitation Environments and Services

Mapping the workshop outputs to the original framework suggested additions and augmentations to the framework (see Supplemental Material 5). The fundamental objectives, describing what is ultimately most important to achieve in inpatient stroke rehabilitation environments, remained unchanged from the original framework (Lipson-Smith et al., 2019). Eleven of the original 15 means objectives (i.e., the objectives considered instrumentally important to achieving the fundamentally important things) remained unchanged, with four of the means objectives receiving a definitional expansion or update. Four new means objectives were added, resulting in a total of 19 means objectives (see Table 5). The updates and additions primarily related to the service (model of care, clinical practices, etc.) as the original framework had focused primarily on the physical environment.

**Table 5.**
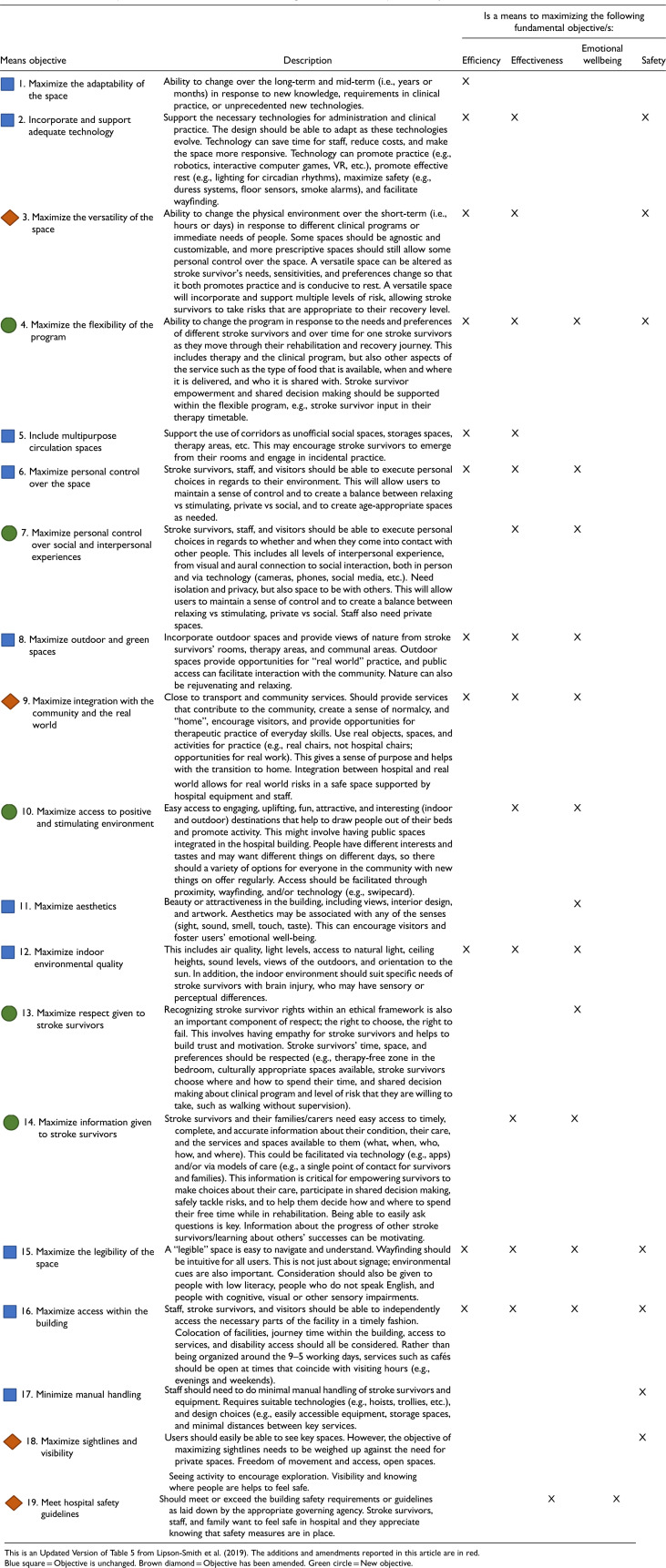
The 19 Means Objectives That are Instrumental for Achieving the Fundamental Objectives in Inpatient Stroke Rehabilitation Environments.

### Engagement 2: Speculative and Visionary Descriptions of Stroke Rehabilitation Environments and Services

Ten practicing healthcare architects and three healthcare architecture academics were involved in Engagement 2. In these workshops, the 28 virtual cards developed from actionable outputs in Engagement 1 and associated means objectives were combined as a series of narrative scenarios—stories that told potential, imagined experiences, or “experiential futures” (Candy & Dunagan, 2017). The architect attendees collaboratively developed 10 narrative scenarios that combined ideas from the 28 virtual cards and mapped to the means objectives defined in Engagement 1 (see Supplementary Material 6). Of the 10 scenarios, five were defined by a physical space or destination on the ward—(1) Bedrooms; (2) Therapy spaces; (3) Communal spaces; (4) Corridors; (5) Staff spaces–and five were service-based–(6) Wayfinding; (7) Admission; (8) Discharge; (9) Empowered and informed; and (10) Levels of risk.

**Figure 4.**
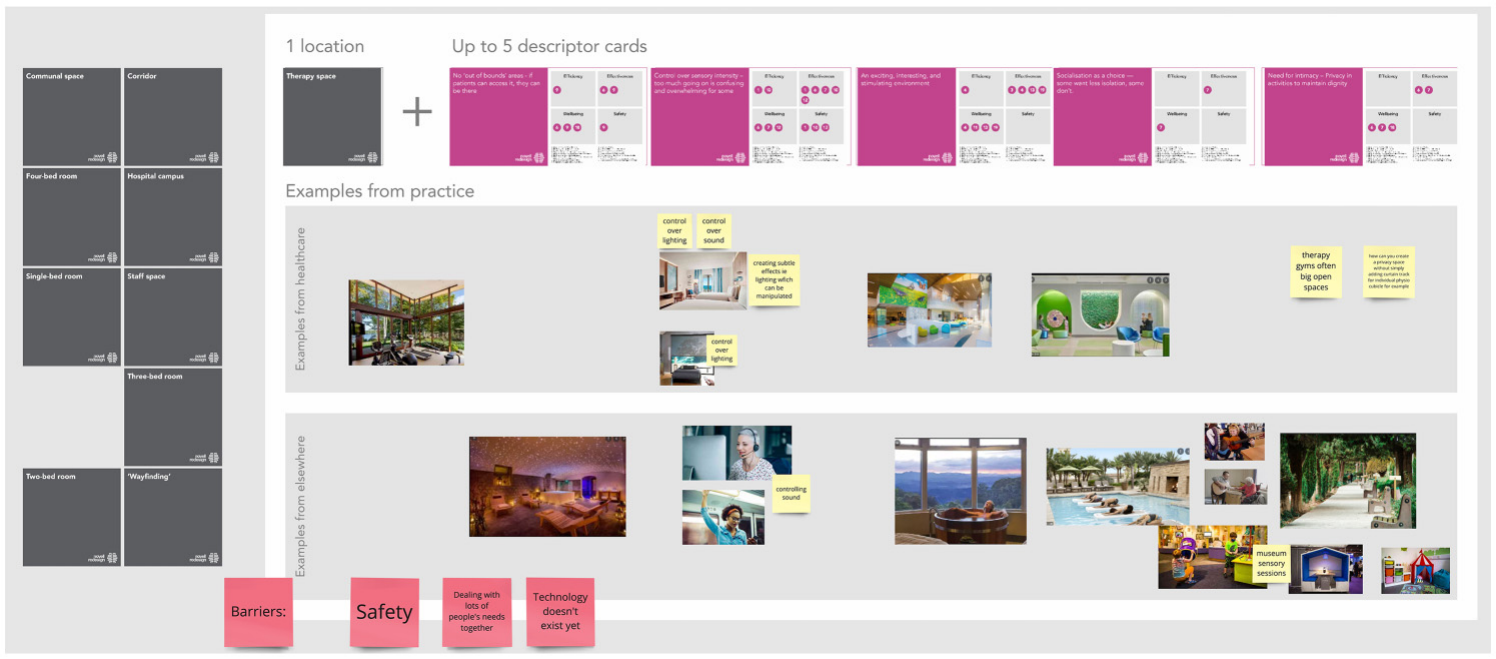
Screenshot of the shared online workspace (Miro board) from Engagement 2, showing an overview of how the scenario cards (actionable outputs aligned with objectives) and real-life design precedents or visionary design examples were combined by attendees to create a narrative scenario for therapy spaces. The detail in this example is not intended to be legible, rather, the figure shows an overview of how different elements were combined to create a narrative scenario, which provided a basis for a design brief.

Figure 4 shows an example of how the virtual cards were combined by co-researchers to develop the therapy spaces scenario. Together, the 10 scenarios describe a visionary stroke rehabilitation environment and service and form the basis of an experiential design brief and speculative description of the stroke rehabilitation facility of the future. In later stages of the project (beyond the scope of this publication), these were further refined with specific technological explorations and phenotype/persona descriptions in a “Rehabilitation Futures” document. An initial draft of this document was developed incorporating the data from Engagement 2, together with relevant outputs from Engagement 1. Asynchronous co-researcher input was then sought (Davis et al., 2021), with the narratives in this document reviewed and added to by the Engagement 2 workshop attendees and the wider co-researcher group. The resulting “Rehabilitation Futures” document is provided in Supplemental Material 7.

*Together, the 10 scenarios describe a visionary stroke rehabilitation environment and service.*

Two of the scenarios–“Bedrooms” and “Levels of risk”–are described in detail below to provide examples of the type of content included in the scenarios. These two scenarios were considered particularly important by stakeholders (see Engagement 3 results below). The remaining eight scenarios are described in Supplemental Material 6.

#### “Bedrooms” Scenario: A Single Room That Achieves the Benefits of a Shared Room, or Vice Versa

Stakeholders identified positives and negatives to both single and shared bedroom designs, sparking the idea that there might be a third option: a hybrid which incorporates the benefits of both a single bedroom (privacy and control) and a shared bedroom (socialization and safety). Whether single or shared, every bedroom should provide privacy and opportunities for social interaction. Privacy promotes emotional wellbeing (dignity, a sense of empowerment and ownership over the space), allows for intimacy with family, and a space for quiet, self-directed therapy. On the other hand, having other people around can promote incidental social interaction, learning from other stroke survivors, and safety through visual connection and proximity (Shannon et al., 2018). It is important that stroke survivors are encouraged to spend time outside of their bedroom and feel connected to the outside world, but that they still have opportunities to engage in a positive and stimulating environment to promote activity and practice even when in the bedroom. Bedrooms should be adaptable and versatile, providing different levels of social interaction, risk, and privacy for different stroke survivors and different stages of the rehab journey and at different times of day. In the bedroom, stroke survivors should feel personal control over the space and over their social and interpersonal experiences.

*Stakeholders identified positives and negatives to both single and shared bedroom designs, sparking the idea that there might be a third option: a hybrid which incorporates the benefits of both a single bedroom (privacy and control) and a shared bedroom (socialization and safety).*

#### “Levels of Risk” Scenario: A Facility That Allows Stroke Survivors Access to Appropriate Levels of Risk, Depending on Individual Preferences and Needs

Stakeholders emphasized the “need to balance risk, not be too risk averse, allow people to fail safely, and have the practices in place to manage and grow through these experiences” (Engagement 1, Workshop 4). Some stroke survivors appreciate access to risk and real-world practice for learning and recovery outside of their formal therapy sessions, while others, often those earlier in their recovery, need and want to avoid risk. A fun, stimulating, and positive environment, with variety and change, can be a motivating challenge, but it should feel safe and supported – this is, after all, the point of being in hospital, rather than out in the community.

Stakeholders suggested that the environment should incorporate opportunities for different “zones of recovery”; different levels, or grades, of risk tailored to the person's needs or preferences. Technology could be incorporated to simulate risk (e.g., virtual reality experiences), or the facility could be integrated with the community and the real world, thereby inevitably bringing different levels of risk into the building. The “zones of recovery” could be analogous to ski runs: “green” zones for people early in their recovery journey who need a lot of support (e.g., supervised practice in a gym), through to “black” zones reserved for those who want an extra challenge before discharge (e.g., out of hours gym access, outdoor access, traveling independently to the café). Stroke survivors should be allowed to choose which zones they can access, as long as it is safe for them to do so, showing trust and respect and giving a sense of empowerment which is important for motivation and emotional wellbeing. Technology could be used to allocate stroke survivors access to designated risk levels (e.g., smart watches for tracking; doors that open for some people but not others).

*Stakeholders suggested that the environment should incorporate opportunities for different “zones of recovery”; different levels, or grades, of risk tailored to the person's needs or preferences.*

### Engagement 3: Prioritized List of Scenarios

A panel of co-researchers who had been part of Engagement 1 were invited to complete the online prioritization task to rank the scenarios (Engagement 3). Twenty-four co-researchers, including six representatives from each of the four target disciplines (academics; architects/designers; clinicians; and stroke survivors) completed this task.

The Bedrooms scenario was ranked as most in need attention, while Staff spaces were ranked as the least (see Table 6). Levels of risk and Therapy spaces both achieved an equal ranking, just below Bedrooms. The top six scenarios (Bedrooms, Levels of risk, Therapy spaces, Empowered and informed, Communal spaces, and Corridors) all achieved similarly high rankings, with very little difference between the ranking scores. Short answer entries indicated the reasoning behind respondents’ rankings, for example, suggesting that Staff spaces was ranked lower, not because it was not important, but because it was perceived as already receiving attention in healthcare design (see Table 6 and Supplemental Material 8).

**Table 6.** The 10 Scenarios That Together Illustrate a Speculative and Visionary Future for Stroke Rehabilitation Environments and Services.

Overall rank	Scenario title	Brief description	Illustrative quotes from short-answer responses
1	Bedrooms^a^	A single room that achieves the benefits of a shared room, or vice versa	“Bedrooms are really important because of the current push for single bed rooms, as the solution for all ills—too simplistic … Bedrooms [also] become important when we think of social isolation and falls as part of safety.” Researcher 1
2^b^	Levels of risk^c^	A facility that provides stroke survivors with access to desired level of risk, allowing for individual needs and flexibility.	“Levels of risk is important for safety, a feeling of growing independence and confidence, experimentation and reward, safety nets” Stroke survivor 1 “Process and culture impact on psychological safety and ability to plan and take suitable risks. The lack of real-world scenarios make it an unsafe transition to home. The design makes it hard to support practice of real tasks and we are too risk adverse, thus stop people from having a go, when they need to give it a measured try.” Clinician 1
2^b^	Therapy spaces^a^	Therapy spaces that provide opportunity for supported challenge—promoting practice in the context of real-world activities, both indoor and outdoor, while also providing the convenience and safety of being in hospital.	“Therapy spaces at present are just differently named gyms and offices, they could be so much more … Therapy spaces need to be both flexible where possible and single purpose when not, e.g., a corner of the gym is not suitable for goal setting, it is impossible to concentrate on activation of arm muscles within a loud and disordered environment” Stroke survivor 1 “the challenge of integrating hospital and the outside world in in new ways - not just by doing more home visits! The interim staging - not a room that looks like a home - but ideas around small communal households or hotel step down is really interesting and worth exploring further.” Researcher 1
4	Empowered and informed^c^	Communication and information provision so that stroke survivors and their families always know what is available to them and what will be happening next in their care. This relates to the whole facility design, technology, and also the design of the service.	“I feel that if a person and their family are empowered and informed then this will make changes to many of the other contexts, for example, admission, discharge, and risk. Autonomy and empowerment through the rehabilitation process are integral to engagement in rehabilitation and for rehabilitation outcomes. I also think there is a lot of potential for change in this context.” Clinician 6 “Empowered and informed targets a lot of different areas in the facility and covers off how the patient and family may feel putting their feelings as they forefront rather than being targeted upon a specific space or room.” Designer 2
5	Communal spaces^a^	Communal spaces that create an exciting, interesting, and stimulating environment, but also provide opportunities for privacy, and peaceful or spiritual spaces for relaxation. Spaces that are appropriate for a range of cultures without being bland.	“I have ranked these based on my experience of my mother's stroke. The proximity/communication with nursing staff and other patients was one of the most critical for her (as an extravert that loves social interaction with people).” Researcher 6 “Need a flexible environment because not everyone on their rehab journey will want to socialise at all points on their journey, and this is also linked to control. I didn't want socialisation at the start—found discouraging to see others in bad position, just wanted to be active … but did have an experience at the end of rehab where someone who saw me doing a test said ‘you're doing so well’ and that was really encouraging. I like the idea of having different levels of socialisation …” Stroke survivor 6
6	Corridors^a^	Corridors that provide accessible and safe social spaces, retreat spaces, spaces to rest along a journey, and control over sensory stimuli.	“Corridors should be more than places where you wait, are parked, left, travel through … They could provide so many more opportunities to promote wellbeing in terms particularly safety, belonging, socialisation.” Stroke survivor 1 “The prioritisation was driven a lot by visibility and sight lines. What can the staff see/monitor/passively observe especially entries, admissions, discharge, circulation spaces. Having an awareness of the users/family/staff within the facility, where people are, and being able to intervene if someone is upset or struggling.” Designer 2
7^b^	Wayfinding^c^	Wayfinding that functions at multiple levels and adapts for different stages of the rehabilitation journey. This relates to the whole facility design, technology, and also the design of the service.	“I don't think [other spaces] are as much in need of innovation as the connections between spaces, ie corridors and wayfinding, so stroke survivors can have confidence and empowerment to find what they need/want.” Designer 4 “Patient awareness of where they can and cannot be within a facility and having that clearly articulated can help with a sense of safety as well.” Designer 2
7^b^	Admission^c^	A facility and service that eases the process of admission to rehabilitation, that feels instantly welcoming and secure, that stroke survivors and families can easily become familiar with and understand.	“There is huge potential to improve admission & discharge - there is a lot of work needed in these spaces … hard to pick which is first. I think improving “empowered & informed” would improve admission & discharge as well.” Clinician 3 “Admission spaces are important as the first point of interaction with a facility. It's a tricky one. Often patients arrive in an ambulance in a back of house corridor into service lifts due to separation from public entrances. Pending the size of the facility and the number of expected admissions, prioritisation of the look and feel of this space often gets overlooked.” Designer 2
7^b^	Discharge^c^	A facility and service that eases the process of discharge from rehabilitation, that creates a graded transition between hospital and home by bringing the real-world in and expanding the rehab-world out.	“Admission I put as 9 because I don't remember admission, but discharge I put as 4 because it was so badly done, and I was overwhelmed.” Stroke survivor 6
10	Staff spaces^a^	Staff spaces and nurses’ stations that allow staff to communicate easily with each other, provide good line of sign and visibility, while also providing auditory privacy for discussing stroke survivor care.	“I put staff spaces in the middle, as I have experience of the most beautifully designed buildings not functioning as they don't work with the way the staff needs to work in that space, and when this happens the staff use the space differently than how it was intended. In these cases, the theory does not translate to practice, so doesn't have the intended benefits for the patients. This shows the importance of working with all key stakeholders.” Clinician 6 “I was reluctant to put staff spaces at the bottom, as staff often get forgotten in this process, but I felt that staff spaces are getting more traction in acute settings and that is why I didn't prioritise for this purpose.” Designer D2

Ranked in order of how in need they are of attention, from most in need (bedrooms) to least in need (staff spaces). Further illustrative quotes are included in Supplementary Material 8.

^a^ Space-based scenario.

^b^ These scenarios achieved equal rankings. If two or more scenarios achieved equal ranking, the next scenario in the priority list was ranked allowing for the total number of scenarios to be 10. For example, if two scenarios were tied at second place, then the subsequent scenario was given a fourth-place ranking.

^c^ Service-based scenario.

## Discussion

In this manuscript, we have reported the methods and findings of the first phase of a Living Lab project. In line with our aims, we generated specific, actionable, and solution-focused ideas of how to optimize the built environment and the service for inpatient stroke rehabilitation.

The framework defined in Table 5 is an extension and confirmation of previous work (Lipson-Smith et al., 2019), but developed and expanded using different methods, and with different co-researchers, thereby strengthening the validity of our findings. The 19 means objectives in the present framework comprehensively describe *how* to achieve an optimal inpatient stroke rehabilitation environment and service that is efficient and that supports the clinical outcomes of stroke survivors through physical, cognitive, communication and social activity, and the emotional well-being and safety of all users. Critically, our methods encouraged participants to describe actionable, specific design suggestions to meet each of the defined objectives. The inclusion of these 28 actionable suggestions, offered by key stakeholders, is a unique contribution of this study.

*The 19 means objectives in the present framework comprehensively describe *how* to achieve an optimal inpatient stroke rehabilitation environment and service that is efficient and that supports the clinical outcomes of stroke survivors through physical, cognitive, communication and social activity, and the emotional well-being and safety of all users.*

Malterud and Elvbakken's (2020) review of co-researchers in healthcare emphasized the need for research practices to go beyond confirming prior assumptions or prior research through additional stakeholder engagement. The findings of this research demonstrate how carefully constructed methods and ways of working with complex stakeholder groups can enable projects to deliver significant new information and new knowledge about challenges. The use of Design Thinking and generative methodologies for engaging with diverse stakeholders through both Engagement 1 and Engagement 2 delivered significant new insights into the opportunities of healthcare environments. This project demonstrates how divergent and “blue-sky thinking” processes can be integrated into a rigorous and outcome-focused research process, and provides a model for future healthcare innovation projects to engage with complex stakeholder groups.

*This project demonstrates how divergent and “blue-sky thinking” processes can be integrated into a rigorous and outcome-focused research process, and provides a model for future healthcare innovation projects to engage with complex stakeholder groups.*

A key innovation in this process was the use of Speculative Futures (Dunne & Raby, 2013) and Experiential Futures (Candy & Dunagan, 2017) to translate the outcomes from a blue-sky-thinking process (Engagement 1) into a format that can be connected with current and future practice (Engagement 2) and used as the basis for prioritization of innovation (Engagement 3). For policy makers and health planners, this project delivers a series of challenging but important design recommendations that can be incorporated into the briefing and commissioning processes. While speculative and forward-looking, these briefs connect directly to patient outcomes through the means and fundamental objectives in the framework (Table 5). Innovations in healthcare design are rare without traditional forms of evidence linking physical environment to clinical outcomes (Hamilton, 2008, 2014), but this evidence is often lacking of difficult to obtain. The process of connecting design objectives to outcomes provides an alternative vehicle for driving innovation in healthcare design in the absence of other forms of evidence.

Previous research in stroke rehabilitation environments has emphasized the contrasting priorities of, on the one hand, rest and privacy for stroke survivors, and on the other, the importance of stimulation, and physical, communication, cognitive, and social activity in inpatient stroke rehabilitation (Anåker et al., 2024; D'Souza et al., 2021; Kevdzija et al., 2022; Lipson-Smith et al., 2023). Several of the actionable suggestions offered by co-researchers are relevant to this dichotomy between rest/privacy and stimulation, and offer potential design solutions. For example, the suggestions to have “retreat and rest areas within the bedroom,” and “an exciting, interesting, and stimulating environment,” and “socialisation as a choice,” address this issue through stroke survivor empowerment and choice. The contrasting priorities of rest/privacy and stimulation have been highlighted in the debate over the various benefits of single, versus multiple-bed rooms; while single-bed rooms are encouraged in acute care settings, their benefit in stroke rehabilitation has been questioned due to the risk of social isolation and reduced activity (Shannon et al., 2018). Our “Bedrooms” scenario reframes this debate by asking designers to strive to achieve a bedroom that provides the benefits of both a single and shared room; thereby focusing on the objectives that we want the room to achieve (outlined in Tables 4 and 5, e.g., personal control over the space, respect for stroke survivors, a positive and stimulating environment), rather than focusing on the existing alternative design norms of single and multiple-bed rooms. The actionable suggestions in the scenarios, considered alongside evidence from previous literature (Lipson-Smith et al., 2021), provide a reference for designers who are seeking to rethink the single, versus multiple-room dichotomy and consider other options besides the standard single, double, or four-bed room templates that are recommended in healthcare design guidelines.

*Several of the actionable suggestions offered by co-researchers are relevant to this dichotomy between rest/privacy and stimulation, and offer potential design solutions.*

The impact of stroke is heterogeneous, and stroke survivors’ needs will change over the course of their stay in stroke rehabilitation. Several of the means objectives in our framework speak to the need for adaptive and flexible environments and services (see Table 5). The “Levels of Risk” scenario describes an environment where the environment, technology, and service collectively provide stroke survivors with safe access to desired level of risk, allowing for individual needs and flexibility. This concept of graded risk could be extended beyond the physical environment to address the possibility of having different levels of care built into the service, and beyond the hospital environment itself. While inpatient rehabilitation is becoming less common worldwide as more facilities encourage early supported discharge and at-home care (Langhorne et al., 2017), it remains essential for those unable to rehabilitate at home. Further, hybrid models of care, virtual and in-person, are emerging and should be incorporated into all new rehabilitation designs. In all cases, models of care could be aligned to the different levels of risk that occur in different environments, ranging from inpatient care, to transitional care or non-traditional healthcare spaces such as converted hotels or group homes, to rehabilitation at home.

The prioritization process used in Engagement 3, driven by the fundamental objectives and previously described scenarios, enabled participants to move beyond the consideration of individual elements or ideas as being “good/bad,” “achieved/not-achieved,” and to consider options beyond their “favourite” or “preferred” ideas. By presenting participants with scenarios rather than elements or variables, then unpacking the means objectives these scenarios contained, we gained insights into which scenarios, and therefore which means objectives and which locations within the rehabilitation environment were most in need of innovation. This aligns with the Living Lab approach of working with complexity, and contrasts with a traditional model of evidence-based design in healthcare by considering multiple variables together rather than in isolation.

*This aligns with the Living Lab approach of working with complexity, and contrasts with a traditional model of evidence-based design in healthcare by considering multiple variables together rather than in isolation.*

The stakeholders included in this study were primarily based in Australia. Aspects of our findings may therefore be specific to the Australian healthcare context. In Engagement 1, there were fewer stroke survivors than clinicians and designers (Table 2). Stroke survivors were, however, included in every discussion in the workshops and supported to contribute through efforts to reduce power imbalances and adapted activities for people with communication impairments. The methods outlined in this manuscript were highly generative, but the iterative and collaborative process did take time, a luxury that is often lacking in architectural practice and hospital design and procurement. An overarching aim of our Living Lab project is to create strong partnerships between research, evaluation, policy, and practice, and to make all findings freely available, so that learnings from the iterative, collaborative research processes can feed into healthcare design and construction in practice.

The outputs reported in this manuscript provide clearly articulated recommendations for the design of inpatient stroke rehabilitation environments and services, including: bedrooms that achieve the benefits of both a single and a shared room; facilities and services that provides stroke survivors with safe access to desired level of risk, allowing for individual needs and flexibility; therapy spaces that promote practice in the context of real world activities; and communal spaces that create an exciting, interesting, and stimulating environment. The outputs from this phase of the project were limited to written descriptions and 2D representations of design briefs and ideas, but provide the beginnings of a stakeholder-generated evidence-base upon which to drive innovation in stroke rehabilitation environments and services. In future phases of the project, several fully realized design options will be generated in response to the findings reported in the present manuscript, modeled in Virtual Reality, and evaluated by multiple stakeholder groups.

## Conclusions

The Living Lab structure and methodologies used in this research have generated new insights that add to our understanding of subacute rehabilitation environments as well as healthcare environments more generally. The study demonstrates how healthcare environments research can be conducted with complex and distributed stakeholder groups, incorporate a variety of research tools, The study demonstrates how healthcare environments research can be conducted with complex and distributed stakeholder groups, incorporate a variety of research tools, including futures and foresight practices, and partner with co-researchers across both generative and analytical phases.

*The study demonstrates how healthcare environments research can be conducted with complex and distributed stakeholder groups, incorporate a variety of research tools.*

Together, the project co-researchers have described a visionary future inpatient stroke rehabilitation facility where the design of the service and the environment are considered interdependent. The actionable insights, described through experiential scenarios and linked with a rigorous structure of fundamental and means objectives, provide new and interesting pathways for healthcare design. In many instances, the findings provide new perspectives on widely encountered challenges, including balancing privacy and activity and deciding between single and multi-bedrooms.

## Implications for Practice

- Designers of stroke rehabilitation facilities should consider how bedrooms can achieve the benefits of both a single and shared bedroom by designing to the relevant means objectives (e.g., maximising personal control over the space, and maximising access to a positive and stimulating environment, etc.) and the associated actionable design ideas (e.g., providing space to retreat within the bedroom, and ensuring that socialisation is a choice, etc.).
- Designers of stroke rehabilitation facilities should consider how facilities can allow stroke survivors access to appropriate levels of risk by designing for “zones of recovery” and using technology to allocate and support stroke survivors within designated zones.
- Innovation in healthcare environments must consider both the built environment and the service.
- The Living Lab process was successful in integrating perspectives of all stakeholder groups.

## Supplemental Material

sj-docx-1-her-10.1177_19375867251343910 - Supplemental material for Design Ideas for Inpatient Stroke Rehabilitation Facilities: Living Lab Findings Supplemental material, sj-docx-1-her-10.1177_19375867251343910 for Design Ideas for Inpatient Stroke Rehabilitation Facilities: Living Lab Findings by Ruby Lipson-Smith, BA, BSc (Hons), PhD, Aaron Davis, B Mus, B Arch, M Arch, MSD, PhD, Marcus White, B Arch, PhD, Luis Pflaumer, BSc, M Biomed, Julie Davey, BA, BSW, M.Mngt, Leonid Churilov, BSc (Hons), PhD, Anna Fox, B Arch, Natalie Pitt, B Arch, Ciara Shiggins, BSc (Speech Path), PhD, Juan Pablo Saa, BSc OT (Hons), OTD, MPH, PhD, Mark Lam, BSc (Hons), M Arch, PhD, Julie Bernhardt, BAppSci (Physio), PhD, and on behalf of the NOVELL Collaboration in HERD: Health Environments Research & Design Journal

## References

[bibr1-19375867251343910] Anåker A. Kevdzija M. Elf M. (2024). Enriched environments in stroke units: Defining characteristics and limitations. HERD: Health Environments Research & Design Journal, 17(2), 344–359. 10.1177/19375867231224972 38494920 PMC11080395

[bibr2-19375867251343910] Australasian Rehabilitation Outcomes Centre (AROC). (2020, January 10). *The State of Rehabilitation in Australia in 2023.* The AROC Annual Report: State of Nations. https://documents.uow.edu.au/content/groups/public/@web/@chsd/@aroc/documents/doc/uow276767.pdf

[bibr3-19375867251343910] Belton V. Stewart T. J. (2002). Multiple Criteria Decision Analysis: An Integrated Approach. Springer US.

[bibr4-19375867251343910] Bernhardt J. Lipson-Smith R. Davis A. White M. Zeeman H. Pitt N. Shannon M. Crotty M. Churilov L. Elf M. (2022). Why hospital design matters: A narrative review of built environments research relevant to stroke care. International Journal of Stroke, 17(4), 370–377. 10.1177/17474930211042485 34427477 PMC8969212

[bibr5-19375867251343910] Brandt E. (2006, August 1–5). Designing exploratory design games: A framework for participation in participatory design? [Conference paper]. In The ninth conference on Participatory design: Expanding boundaries in design, Trento, Italy. 10.1145/1147261.1147271

[bibr6-19375867251343910] Brause C. (2016). The designer’s field guide to collaboration. Routledge.

[bibr7-19375867251343910] Candy S. Dunagan J. (2017). Designing an experiential scenario: The people who vanished. Futures, 86, 136–153. 10.1016/j.futures.2016.05.006

[bibr8-19375867251343910] Carthey J. (2020). Interdisciplinary user groups and the design of healthcare facilities. HERD: Health Environments Research & Design Journal, 13(1), 114–128. 10.1177/1937586719843877 31010311

[bibr9-19375867251343910] Corbin J. Strauss A. (2014). Basics of qualitative research: Techniques and procedures for developing grounded theory. Sage publications.

[bibr10-19375867251343910] Davis A. Wallace N. Langley J. Gwilt I. (2021). Low-contact co-design: Considering more flexible spatiotemporal models for the co-design workshop. Strategic Design Research Journal, 14(1), 124–137. 10.4013/sdrj.2021.141.11

[bibr11-19375867251343910] D’Souza S. Godecke E. Ciccone N. Hersh D. Janssen H. Armstrong E. (2021). Hospital staff, volunteers’ and patients’ perceptions of barriers and facilitators to communication following stroke in an acute and a rehabilitation private hospital ward: A qualitative description study. BMJ Open, 11(5), e043897. 10.1136/bmjopen-2020-043897 PMC8103362 33952543

[bibr12-19375867251343910] Dunne A. Raby F. (2013). Speculative Everything: Design, Fiction, and Social Dreaming. MIT press.

[bibr13-19375867251343910] Glaser B. G. Strauss A. L. (2017). Discovery of grounded theory: Strategies for qualitative research. Routledge.

[bibr14-19375867251343910] Halawa F. Madathil S. C. Gittler A. Khasawneh M. T. (2020). Advancing evidence-based healthcare facility design: A systematic literature review. Health Care Management Science, 23(3), 453–480. 10.1007/s10729-020-09506-4 32447606

[bibr15-19375867251343910] Hall S. Oldfield P. Mullins B. J. Pollard B. Criado-Perez C. (2017). Evidence based practice for the built environment: Can systematic reviews close the research—practice gap? Procedia Engineering, 180, 912–924. 10.1016/j.proeng.2017.04.341

[bibr16-19375867251343910] Hamilton D. K. (2008). Evidence is found in many domains. HERD: Health Environments Research & Design Journal, 1(3), 5–6. 10.1177/193758670800100302 21161904

[bibr17-19375867251343910] Hamilton D. K. (2014). Research informed design, best practice, and fresh perspectives: Can we all get along? HERD: Health Environments Research & Design Journal, 7(3), 94–97. 10.1177/193758671400700307 24782238

[bibr18-19375867251343910] Hamilton D. K. (2015). Collaborators must work together to share results. HERD: Health Environments Research & Design Journal, 9(1), 107–109. 10.1177/1937586715596188 26338306

[bibr19-19375867251343910] Keeney R. L. (1992). Value-focused thinking: A path to creative decisionmaking. Harvard University Press.

[bibr20-19375867251343910] Kevdzija M. Bozovic-Stamenovic R. Marquardt G. (2022). Stroke Patients’ free-time activities and spatial preferences during inpatient recovery in rehabilitation centers. HERD: Health Environments Research & Design Journal, 15(4), 96–113. 10.1177/19375867221113054 PMC9523820 35850529

[bibr21-19375867251343910] Langhorne P. Baylan S. , & early supported discharge trialists. (2017). Early supported discharge services for people with acute stroke. Cochrane Database of Systematic Reviews, 7, CD000443. 10.1002/14651858.CD000443.pub4 PMC6483472 28703869

[bibr22-19375867251343910] Leminen S. Westerlund M. Nyström A. -G. (2012). Living labs as open-innovation networks. Technology Innovation Management Review, 2(9), 6–11. http://timreview.ca/article/602 https://doi.org/10.22215/timreview/602

[bibr23-19375867251343910] Lipson-Smith R. Churilov L. Newton C. Zeeman H. Bernhardt J. (2019). A framework for designing inpatient stroke rehabilitation facilities: A new approach using interdisciplinary value-focused thinking. HERD: Health Environments Research & Design Journal, 12(4), 142–158. 10.1177/1937586719831450 30799632 PMC6745610

[bibr24-19375867251343910] Lipson-Smith R. Pflaumer L. Elf M. Blaschke S.-M. Davis A. White M. Zeeman H. Bernhardt J. (2021). Built environments for inpatient stroke rehabilitation services and care: A systematic literature review. BMJ Open, 11(8), e050247. 10.1136/bmjopen-2021-050247 PMC8344318 34353805

[bibr25-19375867251343910] Lipson-Smith R. Zeeman H. Muns L. Jeddi F. Simondson J. Bernhardt J. (2023). The role of the physical environment in stroke recovery: Evidence-based design principles from a mixed-methods multiple case study. PLOS ONE, 18(6), e0280690. 10.1371/journal.pone.0280690 PMC10256226 37294748

[bibr26-19375867251343910] Lumivero. (2017). *NVivo* (Version 12) [Computer software]. www.lumivero.com

[bibr27-19375867251343910] Malterud K. Elvbakken K. T. (2020). Patients participating as co-researchers in health research: A systematic review of outcomes and experiences. Scandinavian Journal of Public Health, 48(6), 617–628. 10.1177/1403494819863514 31319762

[bibr28-19375867251343910] Patton M. Q. (2015). Qualitative research and evaluation methods: Integrating theory and practice (4th ed.). Sage Publications.

[bibr29-19375867251343910] Rowe P. G. (1991). Design thinking. MIT press.

[bibr30-19375867251343910] Saa J. P. Lipson-Smith R. White M. Davis A. Yang T. Wilde J. Blackburn M. Churilov L. Bernhardt J. , & on behalf of NOVELL Redesign. (2023). Stroke inpatient rehabilitation environments: Aligning building construction and clinical practice guidelines through care process mapping. Stroke, 54(11), 2946–2957. 10.1161/STROKEAHA.123.044216 37846565

[bibr31-19375867251343910] Sal Moslehian A. Kocaturk T. Andrews F. Tucker R. (2023). The nature of innovation in hospital building design: A mixed grounded theory study. Construction Innovation, 23(4), 792–814. 10.1108/CI-12-2021-0236

[bibr32-19375867251343910] Sal Moslehian A. Kocaturk T. Tucker R. (2020). An integral view of innovation in hospital building design: Understanding the context of the research/practice gap. Building Research & Information, 49(3), 1–16. 10.1080/09613218.2020.1740577

[bibr33-19375867251343910] Sanders E. B.-N. Stappers P. J. (2008). Co-creation and the new landscapes of design. CoDesign, 4(1), 5–18. 10.1080/15710880701875068

[bibr34-19375867251343910] Sanders E. B.-N. Stappers P. J. (2012). Convivial toolbox: Generative research for the front end of design. BIS Publishers.

[bibr35-19375867251343910] Shannon M. M. Lipson-Smith R. Elf M. Olver J. Kramer S. Bernhardt J. (2018). Bringing the single versus multi-patient room debate to vulnerable patient populations: A systematic review of the impact of room types on hospitalized older people and people with neurological disorders. Intelligent Buildings International, 12(3), 180–198. 10.1080/17508975.2018.1548339

[bibr36-19375867251343910] Sjöholm A. Skarin M. Churilov L. Nilsson M. Bernhardt J. Lindén T. (2014). Sedentary behaviour and physical activity of people with stroke in rehabilitation hospitals. Stroke Research and Treatment, 2014, 591897. 10.1155/2014/591897 24772368 PMC3977466

[bibr37-19375867251343910] Stroke Foundation. (2023). *National Stroke Audit – Acute Services Report 2023.* Melbourne, Australia.

[bibr38-19375867251343910] Stroke Foundation. (2024). Clinical Guidelines for Stroke Management. Available at https://informme.org.au/guidelines/living-clinical-guidelines-for-stroke-management. Accessed 19 August 2024, Chapter 5: Rehabilitation

[bibr39-19375867251343910] Thayabaranathan T. Kim J. Cadilhac D. A. Thrift A. G. Donnan G. A. Howard G. Howard V. J. Rothwell P. M. Feigin V. Norrving B. Owolabi M. Pandian J. Liu L. Olaiya M. T. (2022). Global stroke statistics 2022. International Journal of Stroke, 17(9), 946–956. 10.1177/17474930221123175 35975986 PMC9980380

[bibr40-19375867251343910] Utley M. Gallivan S. Mills M. Mason M. Hargrave C. (2007). A consensus process for identifying a prioritized list of study questions. Health Care Management Science, 10, 105–110. 10.1007/s10729-006-9003-6 17323658

[bibr41-19375867251343910] West T. Bernhardt J. (2012). Physical activity in hospitalised stroke patients. Stroke Research & Treatment*,* 2012, 813765. 10.1155/2012/813765 21966599 PMC3182066

