# Supplementary material for: Design Ideas for Inpatient Stroke Rehabilitation Facilities: Living Lab Findings
Source: HERD. 2025 Jul 22;18(4):85–110. doi: 10.1177/19375867251343910 (PMC12480617; doi:10.1177/19375867251343910)
Supplement: sj-docx-1-her-10.1177_19375867251343910 - Supplemental material for Design Ideas for Inpatient Stroke Rehabilitation Facilities: Living Lab Findings [file sj-docx-1-her-10.1177_19375867251343910.docx]

**NOVELL Collaboration Members:**

The below list includes members of the research and management team; members of the Advisory Group; industry partners, people with lived experience, clinicians and other experts who attended NOVELL workshops and/or contributed to evaluation sessions; and the winners of the 2022-23 UIA student design competition. Members who opted out of having their name included in disseminations have been excluded below.

The below list is current as of 18^th^ July 2024.

| **First name** | **Last name** | **Organisation or discipline** |
| --- | --- | --- |
| David | Allison | Clemson |
| Stephanie | Antonopoulous | STH |
| Maryam | Banaei | University of technology Sydney |
| Carloyn | Barnes | Swinburne University |
| Nikki | Beckman | Marchese partners |
| Julie | Bernhardt | Florey |
| Marnie | Blackburn | AusHFG |
| Shari | Blanch | Jacobs |
| Sarah-May | Blaschke |  |
| Jannette | Blennerhassett | Austin health |
| Nick | Boulter | Arup |
| Sue | Bowden | Stroke survivor |
| Kylie | Bower | Health dept, QLD |
| Lilian | Braighi Carvalho | Florey |
| Finn | Butler | Design (wayfinding) |
| Biao | Chen | Beijing University of Civil Engineering and Architecture, China |
| Meng | Chen | Harbin Institute of Technology, China |
| Hsin-Hsien | Chiu | Harbin Institute of Technology, China |
| Simon | Chong | Health dept, VIC |
| Leonid | Churilov | Uni of Melb |
| Johanna | Cooper |  |
| Kate | Copeland | AHDC |
| Shalyce | Corney | AusHFG |
| Sabine | Corsten |  |
| Domenica | Cosentino | Formally MCR |
| Maria | Crotty | Flinders |
| Han | Cui | Beijing University of Civil Engineering and Architecture, China |
| Sarah | D'Souza | Clinician |
| Jonathan | Daly | Architecture (environmental psych) |
| Julie | Davey | Consumer Advisor |
| Aaron | Davis | Florey; Uni SA |
| Kristen | Day | Architecture (research) |
| Jasmin | Day | Swinburne (RA) |
| Alison | de Kruiff | Swinburne |
| Claire | Delaney | Ethicist |
| Marie | Elf | Dalarna |
| Kevin | English | Stroke survivor |
| Karen | English | care partner |
| Natalie | Fini | Clinician |
| Paul | Fink | Stroke survivor |
| Anna | Fox | STH |
| Emma | Gee | Stroke survivor |
| Jan | Golembiewski | Architecture (research) |
| Xiaohui | Guo | Beijing University of Civil Engineering and Architecture, China |
| Kirk | Hamilton | Texas AMU |
| Sam | Harvey | Clinician |
| Kelvin | Hill | Stroke Foundation |
| Tonya | Hinde | Architecture (interior) |
| Weijie | Hu | Swinburne University |
| Tingwan | Huang | Beijing University of Civil Engineering and Architecture, China |
| Nan | Jiang | Harbin Institute of Technology, China |
| Carolyn | Jo | Jacobs |
| Mohsen | Kafaei | Swinburne University |
| Georgina | Karavasil | Formally MCR |
| Katrina | Kenah | Clinician |
| Warren | Kerr | AHDC |
| Rhonda | Kerr | Health Economist |
| Byeongsoo | Kim | Kwangwoon University, South Korea |
| Mark | Lam | Swinburne |
| Nano | Langenheim | Architecture (research) |
| Natasha | Lannin | Monash |
| Luci | Lanyon | Clinician |
| Mehrnoush | Latifi Khorasgani | Swinburne |
| Kim | Leenards | Engineering IT |
| Sarah | Lewandowski | Architecture |
| Cath | Lewin | Jacobs |
| Yutong | Li | Harbin Institute of Technology, China |
| Fei | Lian | Harbin Institute of Technology, China |
| Irina | Lindquist | Stantec |
| Ruby | Lipson-Smith | Florey; Western Sydney Uni |
| Soren | Luckins | Design (wayfinding) |
| Maximilian | Luhrs-Dowd | Swinburne (RA) |
| Rob | Luxford | Vivid Wayfinding |
| Fujia | Lv | Harbin Institute of Technology, China |
| Dominic | Martens | Architecture |
| Louise | Massie | Clinician |
| Emy Sandrine | Masso | National Advanced School of Public Works, Cameroon |
| Manning | McBride | MCR |
| Robert | McGauran | Architecture (research) |
| Thomas | McLachlan | Jacobs |
| Remika | Mito | Florey |
| Natasa | Momcilovic | STH |
| Lea | Moos |  |
| Leanne | Muns | Clinician |
| Baudrel | Nde | National Advanced School of Public Works, Cameroon |
| Toni | Neck | Jacobs |
| Clare | Newton | Architecture (research) |
| Michael | Nilsson | Newcastle Uni |
| Phoung | Nugyen | Formally MCR |
| Yulie | Olsson-White | Jacobs |
| Wen | Ouyang | Beijing University of Civil Engineering and Architecture, China |
| Jeni | Paay | Swinburne |
| Kyeonghyeon | Park | Kwangwoon University, South Korea |
| Brooke | Parsons | Stroke survivor |
| Julia | Paxino | Clinician |
| Alan | Pert | Uni of Melb |
| Tanya | Petrovich | Architecture |
| Luis | Pflaumer | Uni of Melb |
| Natalie | Pitt | STH |
| Almut | Plath |  |
| Rob | Polglase | Architecture (research) |
| Adam | Pustola | Architecture |
| Alyce (Elise) | Raine | Stroke survivor |
| Caleb | Rixon | Stroke survivor |
| Geraint | Rogers | Microbiologist (infection control) |
| Juan Pablo (JP) | Saa | Florey; La Trobe |
| Konrad | Samulis | STH |
| Stefano | Scalzo | DHHS |
| Belinda | Seale | Deakin |
| Michelle | Shannon | Florey; Monash |
| Mardelle | Shepley | Cornell |
| Zuozheng | Shi | Beijing University of Civil Engineering and Architecture, China |
| Ciara | Shiggins | Speech pathologist |
| Eden | Short | Maynard |
| Efterpi | Soporos | Designer |
| Jiayu | Sun | Harbin Institute of Technology, China |
| Yutong | Sun | Harbin Institute of Technology, China |
| Haibo | Sun | Harbin Institute of Technology, China |
| Georgia | Taylor | Monash Health |
| Fiona | Teo | Austin health |
| Martin | Tomko | Engineering IT |
| Steve Wilson Ntakam | Tonguembo | National Advanced School of Public Works, Cameroon |
| Lizette | Tsafack | National Advanced School of Public Works, Cameroon |
| Stuart | Turk | STH |
| Damon | van Horne | Architecture |
| Michel | Verheem | Design (wayfinding) |
| Michael | Walker | Government |
| Steven | Wells | Clinician and landscape designer |
| Marcus | White | Swinburne |
| Kristen | Whittle | Architecture |
| Jack | Wilde | Uni SA |
| Alice | Williams | Felton |
| Tianyi | Yang | Swinburne |
| Heidi | Zeeman | Griffith |
| Zheyuan | Zhao | Harbin Institute of Technology, China |
